# RNA Structure Elements Conserved between Mouse and 59 Other Vertebrates

**DOI:** 10.3390/genes9080392

**Published:** 2018-08-01

**Authors:** Bernhard C. Thiel, Roman Ochsenreiter, Veerendra P. Gadekar, Andrea Tanzer, Ivo L. Hofacker

**Affiliations:** 1 Department of Theoretical Chemistry, Faculty of Chemistry, University of Vienna, Währingerstraße 17, 1090 Wien, Austria; thiel@tbi.univie.ac.at (B.C.T.); romanoch@tbi.univie.ac.at (R.O.); veerendra@tbi.univie.ac.at (V.P.G.); 2 Research Group Bioinformatics and Computational Biology, Faculty of Computer Science, University of Vienna, Währingerstraße 29, 1090 Wien, Austria

**Keywords:** RNA secondary structure, conserved RNA structure elements, RNAz, structural alignment, RNA regulation

## Abstract

In this work, we present a computational screen conducted for functional RNA structures, resulting in over 100,000 conserved RNA structure elements found in alignments of mouse (mm10) against 59 other vertebrates. We explicitly included masked repeat regions to explore the potential of transposable elements and low-complexity regions to give rise to regulatory RNA elements. In our analysis pipeline, we implemented a four-step procedure: (i) we screened genome-wide alignments for potential structure elements using RNAz-2, (ii) realigned and refined candidate loci with LocARNA-P, (iii) scored candidates again with RNAz-2 in structure alignment mode, and (iv) searched for additional homologous loci in mouse genome that were not covered by genome alignments. The 3’-untranslated regions (3’-UTRs) of protein-coding genes and small noncoding RNAs are enriched for structures, while coding sequences are depleted. Repeat-associated loci make up about 95% of the homologous loci identified and are, as expected, predominantly found in intronic and intergenic regions. Nevertheless, we report the structure elements enriched in specific genome elements, such as 3’-UTRs and long noncoding RNAs (lncRNAs). We provide full access to our results via a custom UCSC genome browser trackhub freely available on our website (http://rna.tbi.univie.ac.at/trackhubs/#RNAz).

## 1. Introduction

RNAs fulfill a multitude of regulatory functions in the cell which are often mediated by a particular RNA structure. Functional—and therefore evolutionarily conserved—RNA structures are thus abundant throughout the genome, both in the form of structured noncoding RNAs [1], as well as *cis*-regulatory structures within larger transcripts, such as messenger RNAs (mRNAs). To date, these functional elements remain poorly annotated, since they cannot be easily identified by high-throughput methods.

One valuable experimental approach to fill this gap is structural probing of the whole transcriptome, as reviewed by Choudhary et al. [2]. An overview over different experimental protocols is also given by Saus et al. [3] in Table 1 of their paper. Structure probing has been applied to mouse successfully [4]. Such approaches suffer from the limited abundance of many transcripts, which leads to missing data at many nucleotide positions. Furthermore, by design, structural probing experiments only give information about the structuredness of the transcriptome and not about the functional importance of those structures. RNA duplex experiments show short- and long-range base pairs in the mouse transcriptome [5].

A number of computational methods have been proposed that identify functional RNA structures based on their evolutionary conservation, such as QRNA [6], RNAz [7,8], EvoFold [9], and CMfinder [10]. All of these methods, including our contribution, focus on local structures due to computational limitations.

RNAz uses a support vector machine (SVM)-based approach to classify alignment windows as structurally conserved RNA based on structure conservation and covarying base pairs, as well as the thermodynamic stability of the predicted structure. Evofold uses stochastic context-free grammar to model sequence variation of structured RNAs along a phylogenetic tree. CMfinder is based on covariance models (CMs). Covariance models are “probabilistic models of the conserved sequence and secondary structure of an RNA family” [11], which extend the hidden Markov model to describe sequence pattern of an RNA and secondary structure. While CMs, such as those found in Rfam [12], are built from known RNA families, CMfinder goes one step further by creating covariance models directly from a set of (orthologous) sequences with unknown structures, similar to motif discovery in sequences. An iterative process is then used to optimize the CMs and corresponding alignments.

A few genome-wide screens for conserved RNA structures in vertebrate genomes have been performed using these tools, including [13,14,15,16]. However, all of these screens used human as a reference genome and are therefore of limited use when working with nonprimate species.

In this contribution, we concentrate on the mouse genome as the most important vertebrate model organism, and present a novel screen for functional RNA structures in genome-wide alignments of mouse with other vertebrates. We provide a database of the predicted functional elements for easy use in genome browsers. Furthermore, we introduce a novel and improved pipeline for genome-wide screens using RNAz-2 [8] that employs structural realignment for improved prediction accuracy. While almost all previous screens were restricted to small subsets of the genome, such as the most conserved 5% [13] or the Encyclopedia of DNA Elements (ENCODE) selected regions in Washietl et al. [14], Torarinsson et al. [15], here, we analyzed all alignable regions of the mouse genome, including repetitive regions [17]. There is evidence from multiple studies that the vast majority of the genome is transcribed at some point or under certain conditions, albeit with low abundance [18]. It is therefore most convenient to perform such screens on the whole genome, as opposed to the transcriptome.

## 2. Results

### 2.1. Identification of Structured RNAs

**Figure 1:**
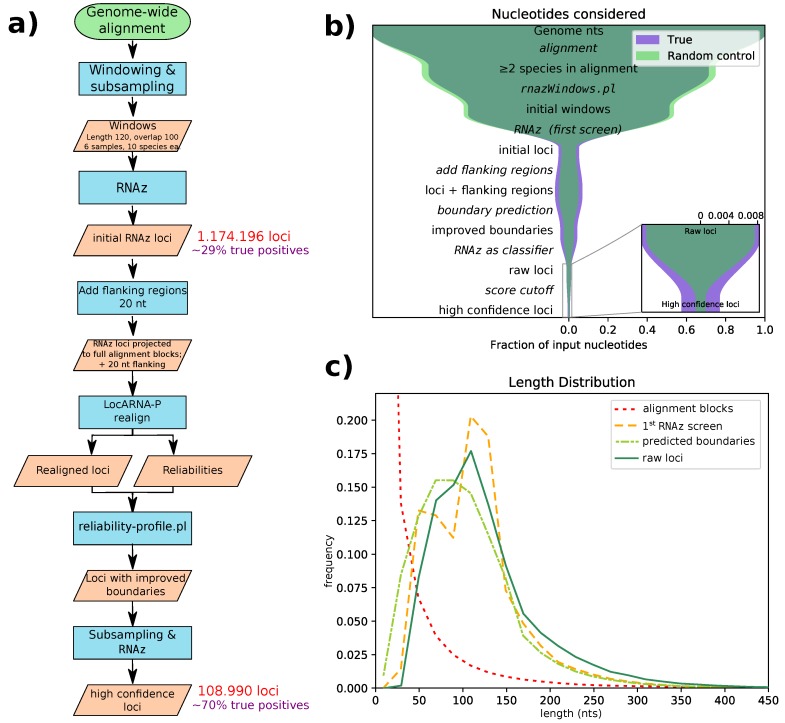
(**a**) A flowchart for our computational pipeline. (**b**) Number of nucleotides considered at every step of the pipeline as a fraction of the number of nucleotides in the genome. The inner (green) funnel is the random control used to estimate the false discovery rate. (**c)** The length distribution of the alignment blocks (red) and our hits at different points of the pipeline.

**Figure 2:**
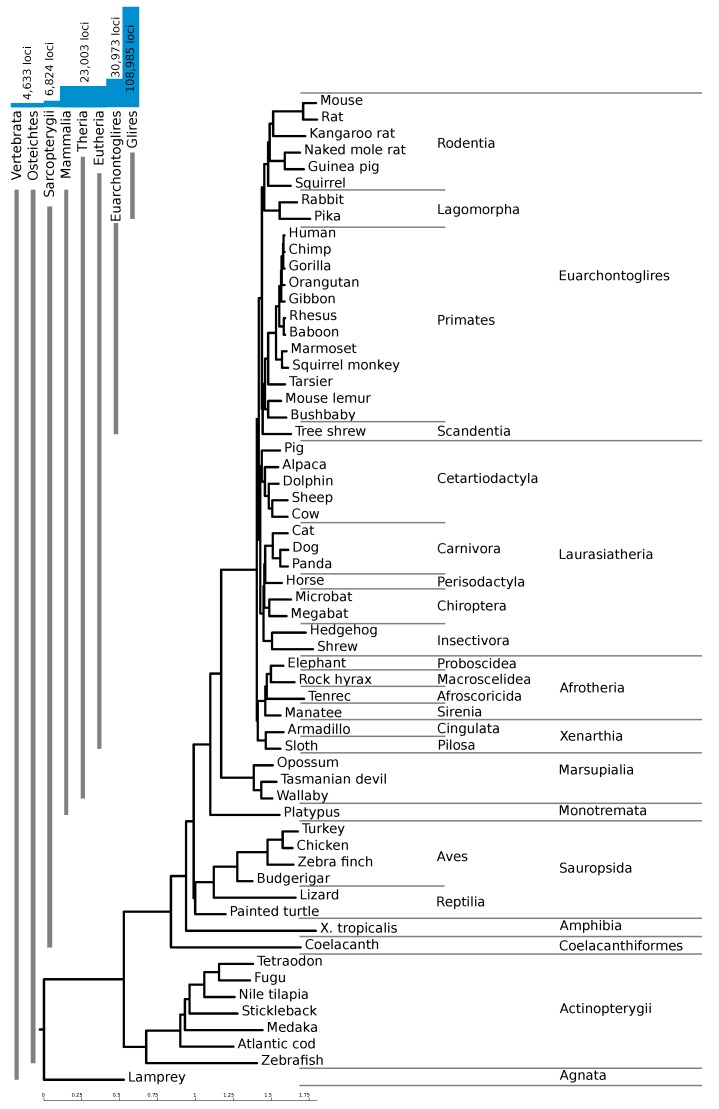
Phylogenetic tree of the 60 vertebrate species included in our screen, and number of high-confidence RNAz loci at different taxonomic levels. The tree corresponds to the one used for the multiz alignments provided by the University of Santa Cruz (UCSC) genome browser [24].

Starting from an alignment of 59 species against the mouse genome [17,19,20], we performed screening and filtering steps to identify genomic regions with conserved RNA secondary structure (see Methods). At its core, our approach is based on RNAz [7,8], but it uses a novel pipeline with several improvements over previous screens. We used RNAz-2 [8], which offers a dinucleotide background model for the calculation of relative thermodynamic stability (*z*-score), as well as a choice of two SVM decision models suitable for sequence alignments and structural alignments, respectively. Our novel pipeline starts with a conventional window-based screen of the input alignments similar to the one described by Washietl and Hofacker [21]. This means that filtered input alignment blocks (see Methods) are cut into windows of size 120 nts (stride 20), and windows with more than 10 species are subsampled to create five alignments of 10 species each. These windows are then classified by RNAz. In contrast to the original pipeline, however, we mitigate some flaws of this first RNAz screen by introducing additional realignment and re-evaluation steps. We use LocARNA-P [22,23] for structural realignment and boundary prediction of the full alignment at the initial loci identified by RNAz, including flanking regions. A second run of RNAz then classifies the realigned loci with improved boundaries, again after subsampling. See Figure 1a for a flowchart of our pipeline. With this pipeline, we identified 108,985 high confidence loci, with an average length of 131 nucleotides. The majority of these loci are confined to regions only alignable within the clade Glires (as shown in Figure 2).

All genome-wide screening approaches for structured RNA suffer from false positives due to the large number of input alignments analyzed. Therefore, our pipeline aims to successively reduce the number of false positives in each step (see Figure 1b). We estimated false discovery rates (FDRs) by applying our pipeline to screen the decoy alignments obtained by randomizing parts of the the original input alignments. According to these estimates, our initial screening step has a fairly high false discovery rate of roughly 70% (in terms of nucleotide coverage). This is comparable to the FDR of a previous screen with RNAz version 1, analyzing the ENCODE selected regions in a human-based alignment [14]. However, by structurally realigning these initial loci, followed by a second classifications step, we significantly reduced the FDR. Finally, we defined a high-confidence subset with an overall FDR of 30% of the loci or 23% of the nucleotides. The FDR depends, of course, on the quality of the input data. As expected, it is higher for alignments with only two species, as well as alignments with high sequence similarity and thus little covariation signal. In contrast, alignments with 3–6 species and low mean pairwise identity exhibit an FDR of roughly 15% (see Supplementary Figure S5).

Structural realignment allows the correction of errors in the input alignment, which become a serious problem below about 60% sequence identity [25], and, consequently, it improves our prediction accuracy for regions with low sequence similarity. However, the realignment step in our pipeline serves additional purposes. We have previously identified the sliding window approach, which slices long input alignments into overlapping windows of 120 nt, as a major shortcoming. If a conserved structure is cut by a window boundary, it clearly cannot be correctly detected, leading to poor sensitivity. On the other hand, using a smaller stride and thus more windows will increase the false discovery rate. LocARNA-P [23] allows the computation of a local structural alignment reliability and uses this to predict the boundaries of a conserved structure. The second RNAz evaluation uses these predicted boundaries, avoiding the need for sliding windows. An artefact of the sliding window approach is seen in the length distribution of the initial RNAz screen’s loci (Figure 1c, yellow dashed line), which has two peaks: one for alignment blocks shorter than the window size and one for overlapping windows. We managed to eliminate this artefact by adding flanking regions and performing boundary prediction after realignment. Flanking region length was set to 20 nt, since this roughly compensates for the shrinkage of loci through boundary prediction. The subsequent RNAz evaluation slightly shifts the distribution of raw hits towards longer loci, a fact that we find desirable, since very short loci tend to contain only generic structures, like single hairpins. Such loci carry little information and thus might be unreliable, especially if they are found in only two species.

Using this novel pipeline and the identified set of high-confidence loci, we now have reliable data which can be used to investigate biological questions.

### 2.2. Distribution of Hits throughout the Genome

**Figure 3:**
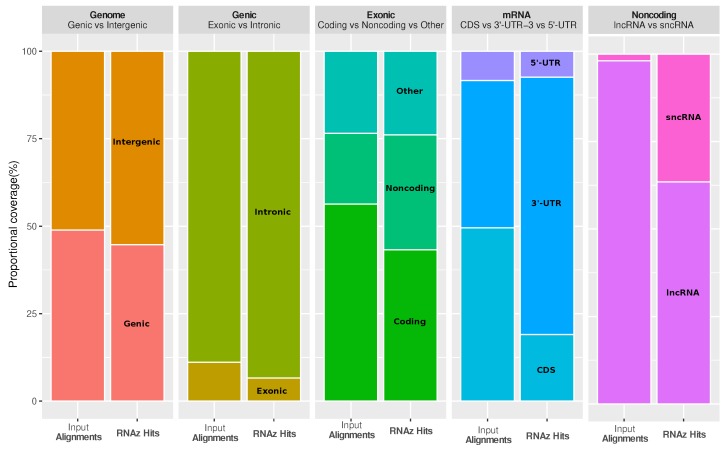
Coverage of different genomic contexts by input alignments and high-confidence loci, respectively. High-confidence loci are slightly depleted in genic vs intergenic regions and in exonic vs intronic regions. They are strongly enriched (1.6-fold) in noncoding exons, which, in turn, is due to the enrichment in the 3’-untranslated regions (3’-UTRs) (1.7-fold) of messenger RNAs (mRNAs). Short non-coding RNAs (sncRNA) contain the classic highly structured small RNAs, such as microRNAs and small nucleolar (snoRNAs), and are strongly enriched compared to long non-coding RNAs (lncRNA). CDS: Coding sequences.

It is interesting to ask how functional RNA structures are distributed over different genomic contexts, such as intergenic region, introns, and spliced transcripts. The most striking observations are that conserved RNA structures are enriched in the 3’-untranslated regions (3’-UTRs) of protein-coding genes, as well as in regions annotated as short noncoding RNA (ncRNA), while coding sequences (CDS) are depleted (see Figure 3). The enrichment in 3’-UTRs is consistent with the fact that these regions carry many *cis*-regulatory elements.

**Figure 4:**
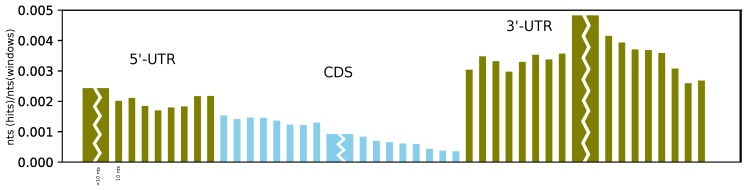
Profile of coverage by RNAz hits at different positions in the mRNA (olive: UTR, light blue: CDS). We plotted the number of nucleotides in RNAz hits at the given position over the number of nucleotides in the initial windows. Each narrow bar corresponds to a bin of 10 nucleotides. Only UTR and CDS annotations, which were long enough to cover all bins, were used. The slightly elevated coverage at the last two 5’-UTR bins may be an artefact of the 20 nt flanking regions, where alignments in the CDS are extended slightly into the 5’-UTR. It is visible here, since (due to better alignability) there are more aligned nucleotides in the CDS than in the 5’-UTR.

Furthermore, on average, we found more structures at the beginning of the CDS than at its end, and we observe a sharp and dramatic increase in structuredness directly after the stop codon (see Figure 4). This can be compared to the probing experiments of Incarnato et al. [4], which showed a significant reduction in probing reactivity (indicating strong base pairing) in the first 50 nucleotides of the 3’-UTR, as well as in the region upstream of the Kozak sequence, compared to the CDS. While we also observe a higher coverage of RNAz hits upstream of the start codon, the effect is less pronounced than in the 3’-UTR. Moreover, the better alignability of the CDS compared to the UTR might have an effect on these results.

While repetitive regions of the genome are often neglected, they have been implicated as a source for the evolution of novel ncRNAs. We therefore looked at the prevalence of conserved structures in repeat regions identified by RepeatMasker [26,27] (see Table 1). The repeat classes with the highest enrichment are simple repeats, satellites, repeats related to structured RNA and short interspersed nuclear element (SINE) repeats, whereas long interspersed nuclear element (LINE) repeats are depleted. The enrichment of repeats was consistent across all genomic partitions (see Supplementary Figure S8). We have recently reported the effects of inverted SINE repeats (iSINEs) on mRNA abundance [28], possibly by folding back on one another and forming long rod-like structures. However, only about 1% of our high-confidence loci contain iSINEs that are close enough to form local structures, suggesting that iSINEs are not a major source for repeat-associated structure elements in our screen.

The high enrichment of satellite repeats is mostly due to the IMPB_01 repeat [29], which has a 12-fold enrichment. The vast majority of our hits overlapping IMPB_01 contain only two species. An example of a locus with support from covarying base pairs overlapping several repeat classes, including an IMPB_01 satellite repeat, is shown in Supplemental Figure S6.

Among the simple repeats, those which contain complementary nucleotides in the repeated region show the highest enrichment, led by CAUG (24 times enriched), as well as UA, UAUAUG, and CAUAUA (all are 18 times enriched). These repeats (or their reverse complement) are complementary to themselves on the RNA level and can form very stable structures. In this case, the conserved structure may be a consequence of the sequence pattern, rather than a consequence of selection pressure on the structure level.

As expected, we found an enrichment in transfer RNAs (tRNAs) and their pseudo-genes. We found 109 loci in regions identified as possible pseudo-genes by tRNAScan-SE [30] and 80 loci in regions identified as tRNA. This translates to an enrichment of 1.7 for pseudo-genes (calculated at the nucleotide level) and a strong enrichment of 6.3 for true tRNAs. While pseudo-genes are thought to evolve without selection pressure, some of them apparently retain remnants of the conserved cloverleaf structure. Indeed, by manual inspection, we found only a few compensatory mutations in tRNA pseudo-genes.

### 2.3. Gene Ontology Specific Enrichment of RNAz Hits

Many of our structured elements are associated with protein-coding genes. This allows us to relate the prevalence of conserved structures with the annotated function of the protein product, and to ask which classes of protein-coding genes are more likely to be regulated at the RNA level. We therefore performed an enrichment analysis for the RNAz hit coverage across protein-coding transcripts associated with various gene ontology (GO) terms. While the 5’-UTRs of the protein coding genes did not show a significant enrichment for RNAz hits, 3’-UTRs were significantly enriched. Moreover, this enrichment was correlated with specific GO terms. A similar but less pronounced enrichment pattern was also reflected in the coding regions. For example, 3’-UTRs of protein-coding genes annotated with the Molecular Function GO term (see Table 2) “binding” were significantly enriched for RNAz hits, both in terms of coverage and number. This suggests that 3’-UTR structures may serve as binding sites or modulate binding activity of the mRNAs [31]. At the same time, a significant depletion of RNAz hits was observed in 3’-UTRs of coding genes annotated as “olfactory receptor” activity or “G-protein coupled receptor” activity. Indeed the mRNAs of the olfactory receptor (*Olfr*) genes were previously identified to have dramatically fewer predicted secondary structures and a higher average AU-content [32].

Similarly, we observed enrichments and depletions of GO terms representing Biological Processes (see Supplementary Table S1) or Cellular Components (see Supplementary Table S2). Particularly noteworthy is the ∼2-fold enrichment of the RNAz hits in the 3’-UTRs of protein-coding genes annotated to the “nervous system development” process. mRNAs of genes in this class are known to experience activity-dependent trafficking to dendrites and local translation [33], which is regulated by 3’-UTR elements.

### 2.4. Identification and Distribution of Structural Homologs

Of particular interest are regulatory structures that occur in several transcripts. In order to identify such RNA families, we built covariance models (CMs) for a subset of 14,119 high-confidence loci with at least three species and a maximum length of 200 nt (see Methods). We then scanned the entire (unmasked) mouse genome, yielding over 11 million significant hits (*E*-value $<10^{-5}$). While 83% of all CMs yield no more than five hits, only 17% of the original CMs account for the overwhelming majority (99%) of hits. This discrepancy can be attributed to the fact that most of the structural homologs stem from highly structured repeat elements that can be found frequently throughout the genome. Among the hits outside repeat regions, we found a high number in intergenic regions of unknown functions. It is tempting to speculate that those structure elements represent yet unannotated ncRNAs or degenerated repeats.

Using the significant hits, we furthermore investigated whether structure elements specifically occur in specific genomic contexts. In contrast to repeat-associated CMs, we observed that the majority of CMs derived from non-repeat loci yield only 1–2 hits in the full genome, usually including their own source sequence. This indicates that many high-confidence loci describe structural features that are unique for their respective gene.

**Figure 5:**
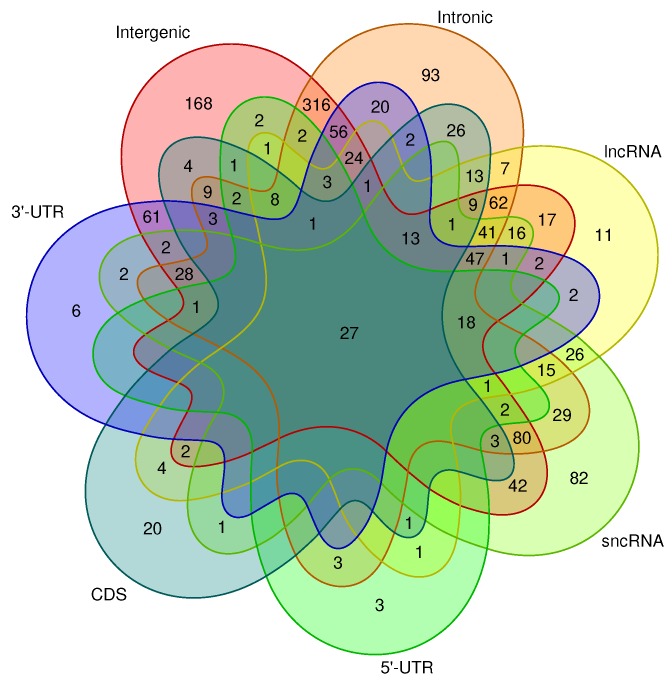
Total number of unique, non-repeat-associated covariance models (CMs) that hit in different genomic intervals. Only CMs with more than two hits were taken into account. Out of 1444 CMs with at least two hits, we observed that only 27 are found among all genomic intervals. Three-hundred-sixteen CMs hit intergenic and intronic regions, while 168 and 93 CMs hit only intergenic or intronic regions, respectively. Eighty-two models exclusively hit short noncoding RNAs (sncRNAs), and a combined 151 models hit sncRNAs and intergenic or intronic or both. Out of all CMs, only 3, 20, and 6 are found exclusively in the 5’-UTR, CDS, and 3’-UTR, respectively. In addition, we found that many CMs give hits in a multitude of different genomic contexts. lncRNA: long non-coding RNA.

Looking at the distribution of hits among different genomic contexts for non repeat-associated CMs with at least two hits (as shown in Figure 5), we found a large number of models to hit in intergenic and/or intronic regions, which is not surprising, since these regions constitute the majority of the genome. It is worth noting that six CMs exclusively hit the 3’-UTR of genes, suggesting that the structures modeled by those CMs might have a potential regulatory function.

In total, 20 CMs exclusively showed hits in the CDS, more than 3 times the number of CMs hitting exclusively 3’-UTRs. However, 61 CMs only hit the 3’-UTR, as well as intergenic regions, while only four CMs are specific for CDS and intergenic regions.

### 2.5. Conservation

As expected, a large number of predicted elements are specific to rodents. Despite the use of a stricter RNAz score cutoff for high confidence loci with only two species in the alignment (see Methods), 74% of our high-confidence hits are only conserved in mouse and one other species, mostly mouse and rat. Figure 2 shows the number of loci conserved within different clades. The vast majority of the high-confidence loci are confined to Glires, underlining the importance of providing a mouse-based screen. About 23,000 loci (21%) are conserved throughout the mammals, and an additional 8000 are conserved between rodents and primates.

### 2.6. Sensitivity

To estimate the sensitivity of our approach to different types of structured RNAs, we investigated the number of annotated small non-coding RNAs in our results. Table 3 shows that we have a good recovery rate for micro RNAs (miRNAs), while our recovery rates for small nuclear RNAs (snRNAs) and small nucleolar RNAs (snoRNAs) are lower, although this still corresponds to an impressive enrichment compared to the rest of the alignment. For the latter two classes, we lost a majority of candidates in the initial RNAz screening step (data not shown). Within snoRNAs and small Cajal body-specific RNAs (scaRNAs), we have a higher sensitivity for HACA-box RNAs, which is expected. We did not find any U6 snRNA, which is not surprising, considering that the predicted consensus structure of this spliceosomal compound shows only a few base pairs, and that U6 can give rise to pseudo-genes [34].

Additionally, it can clearly be seen that we miss more than half of the annotated RNAs because they are split between several alignment blocks. Obviously, if a structural element, such as a hairpin, is divided into two alignment blocks, RNAz cannot find any base pairs and thus does not correctly classify the RNA.

### 2.7. Comparison to Similar Screens

We provide a list of high-confidence predictions of structured RNA loci centered on mouse. Since all previous screens of vertebrate genomes used human as the reference genome, the overlap between their loci and our predicted loci is necessarily small. Of particular interest is the very recent screen of Seemann et al. [16], which uses an unrelated method, CMfinder. Of the 30,973 high-confidence loci that are not specific to Glires, 2220 overlap at least 50% of a locus (i.e., after merging overlapping hits) from Seemann et al. The lack of a larger overlap is not surprising, since RNAz and CMfinder have the best sensitivity for different alignment characteristics [15]. The median length of CMfinder loci, for example, is 59 nt, close to the minimum length for RNAz. We therefore argue that the low overlap is due to limited sensitivity, rather than high false positive rates.

### 2.8. Computation Time

Once the whole pipeline was set up and tested, the computation of the screen took almost 3 months wall-clock time on our in-house cluster with a total of 1304 cores on 33 nodes, and on 96 private cores on the Vienna Scientific Cluster. For the first step of the pipeline, File-IO was limiting, and we used only one physical node (up to 48 cores) per chromosome as a practical way to limit file access over the network. With this setup, the first step took 11 days. The realignment step with LocARNA took around 2 months wall-clock time for the realignment of both strands. It was the only step where more than 2 GB RAM per core were occasionally used, and thus again not all cores could be fully utilized. Boundary prediction and the second RNAz classification step took less than a day each. Calibration of the CMs and subsequent genome screening took about 2 weeks. Most of the computation time was used by very few CMs originating from loci in highly repetitive regions.

### 2.9. Data Availability

**Figure 6:**
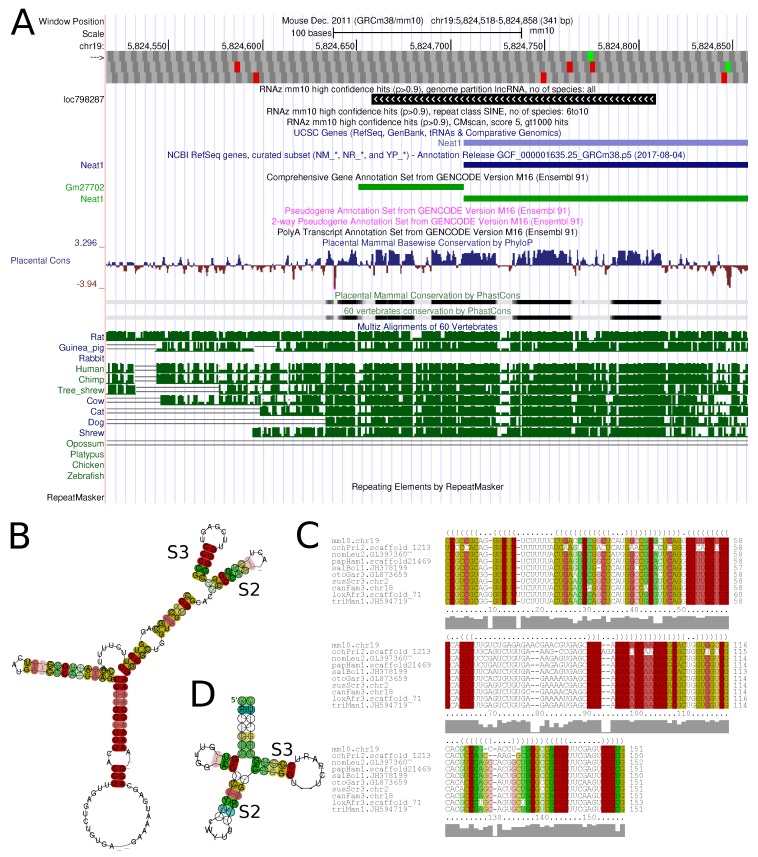
The lncRNA *Neat1* contains a conserved structure element (loc798287) that has some similarity to MALAT1-associated small cytoplasmic RNA (mascRNA, RF01684). We identified a conserved structure element that goes beyond the known mascRNA structure. However, we identified two substructures of mascRNA, stem 2 (S2, panel B and D) and stem3 (S3, panel B and D). (**A**) Browser image of *Neat1* 5’ end; (**B**) Secondary structure plot of loc798287; (**C**) Corresponding alignment with color-coded conservation patterns; (**D**) Consensus structure of mascRNA (RF01684).

We built a publicly available track hub to visualize our results via the UCSC genome browser and made the underlying data easily accessible to the research community. The trackhub ‘RNAz screen by ViennaRNA’ contains three tracks (RNAzGenome, RNAzRepeats, CMstat), each holding a collection of subtracks which can be activated individually. In total, we provided 207 subtracks, allowing users to browse and search through our data in a variety of ways. Figure 6 shows an example of a conserved structure element in the lncRNA *Neat1*.

The track ‘RNAzGenome’ lists all RNAz hits identified in mm10/60 vertebrate alignments and is split into subsets with 2–5 and 6–10 species in the input alignment. These sets are further subdivided into genome partitions. We therefore projected gene elements onto the genome in a hierarchical fashion, thereby creating intervals of unique identity—of particular interest in cases of overlapping transcript isoforms or antisense transcripts. RNAz hits were assigned to genome partitions (min. overlap 60%) and presented in individual subtracks. The ‘RNAzRepeats’ track holds all those hits found in repeat regions, as annotated in the UCSC genome browser ‘repeats’ track. For instance, for the ‘RNAzGenome’ track, a locus must show at least 60% overlap with a repeat element. Tracks are subdivided by repeat class and number of species in the alignment. The ‘CMstat’ track contains all hits of the search with cmscan performed with models built from high-confidence loci. The subtracks here show cmscan hits based on hit quality and number of hits found with each CM model. This way, users can, for instance, easily select frequently occurring high-confidence hits. The trackhub file (http://rna.tbi.univie.ac.at/trackhubs/RNAz/RNAzhub.txt) can easy be loaded into the UCSC genome browser (http://genome-euro.ucsc.edu/cgi-bin/hgHubConnect) and displayed along side with standard or custom tracks to put them in context with other genome annotations and elements. For all RNAz hits, we provided detailed structure information (SVM *p*-value, minimum free energy (MFE) of consensus structure, structure conservation index (SCI), GC content, number of species in alignment) and two images of the structure plot and alignment, both color-coding sequence-structure conservation. All information can be downloaded track by track via the table browser.

## 3. Discussion

We performed a genome-wide prediction of evolutionarily conserved RNA structures in mouse, which we believe will be a valuable resource for researchers interested in regulatory structures, particularly those who use mouse as a model organism. The need for a mouse-centered screen is clearly illustrated by the fact that the majority of predicted loci are not conserved beyond rodents. By including regions only alignable between mouse and rat and carefully adjusting the RNAz score cutoff for loci with only two species, we were able to capture evolutionarily young structures which we would have missed otherwise.

Our improved pipeline, including a realignment step and boundary prediction, tries to ameliorate the effects of windowing and alignment errors. This was achieved to a certain extent, as can be seen from the length distribution of our loci and improved FDR, especially for regions with low sequence identity. Nevertheless, the quality of genome-wide input alignments still has a strong impact on our results. For reasons of computational efficiency, we used structural alignments only in the second stage of the pipeline, where the number of alignments was much smaller. Moreover, realignment could not help in cases where a structured region in the reference genome has been misaligned to a region without the conserved structure in another genome; see [35] for further analysis of genome-wide alignment quality. Note that these types of errors will likewise effect alignment-free methods, such as CMFinder [16].

We chose to use the published 60-way multiz alignment from UCSC as starting point for our analysis instead of creating our own alignment. In addition to the obvious benefit of saving computation time and research time for refining the parameters, there is a more fundamental reason for this choice: this 60-way alignment is the only multiple species alignment for the newest mouse version (mm10) provided by UCSC (except for a recently added four-way alignment of mouse with three Euarchontoglires) and the community standard. By using this alignment, we ensured our results remain comparable to other research projects based on the same alignment.

The use of this published multiz alignment, however, comes with certain disadvantages. The way these multiz alignments are constructed allows for the same sequence of other species to be aligned against multiple regions of the mouse genome. This might be especially relevant for repeat regions without clear orthologs. We found that, for mouse and rat, roughly 90% of the aligned nucleotides are present only once in the alignment. Thus, we do not believe that sequences aligned multiple times caused a noticeable bias in our results. Another effect of the alignment characteristics had much stronger influence on our results: the fact that the alignment contains so many species and tries to include as many species as possible as often as possible leads to fragmentation of the alignment into many, short alignment blocks. Twenty-five percent of the alignment is in blocks with less than 50 columns, which we had to filter out in the beginning of our screen. We did perform merging of alignment blocks after the initial RNAz screen and before realignment, but, for future screens, we recommend merging of alignment blocks before the first filtering. Unfortunately, merging of alignment blocks is not trivial, as there are many parameters that can be optimized. On the one hand, a balance between length of the merged block and the number of species has to be found, since merging usually requires the removal of species from the alignment. On the other hand, the maximum number of nucleotides to be fetched for individual sequences between alignment blocks must be determined. Whenever nucleotides are fetched from the genomic sequence of a species, these nucleotides are not aligned and, thus, an additional realignment step would be necessary, which comes at additional computational cost. Finally, a greedy algorithm might be suboptimal for merging, since there will be cases where an alignment block can be either merged with its upstream neighbor or its downstream neighbor, but not with both. Finally, merging cannot be restricted to only pairs of blocks, since we frequently find the case where longer blocks are separated by multiple blocks of only one or two nucleotides. While we find it highly desirable to test such a merging before the first step, it still requires additional research and parameter tuning. Thus, we have resorted to just merging blocks after an initial classification step with RNAz. This makes a huge difference, because now we can work under the assumption that the blocks indeed belong together. Furthermore, now potential merging only effects less than 1% of the initial loci, thus, one could argue that suboptimal merging parameters do not make much difference in the big picture.

For alignment blocks with more than 10 species, we resorted to subsampling the alignment (see Methods). This limited our ability to assess precisely in which clades of the phylogenetic tree a structure is conserved. Moreover, alignments with many species carry a greater risk of including sequences that do not share a common structure (even though they may exhibit some sequence similarity). In these cases, tools that can split an alignment into subparts with/without a conserved structure would have to be employed [36].

Conserved RNA structures are not distributed uniformly over the genome. Instead, we observed a notable enrichment of conserved structures in 3’-UTRs, while coding sequences contain fewer structures than the genomic background. Some classes of repetitive regions also seem to harbor conserved structures, with satellites showing the strongest enrichment. The mRNAs of different classes of proteins also seem to behave differently: mRNAs and 3’-UTRs of proteins involved in RNA and DNA binding, as well as transcription regulation, seem to be particularly prone to harboring conserved structures, while the mRNAs of G-protein coupled receptors seem to avoid them.

## 4. Materials and Methods

### 4.1. Pipeline for Predicting Structurally Conserved RNA Loci

We obtained multiple sequence alignments of 60 vertebrate species to the mouse genome (mm10/GRCm38, December 2011) from the UCSC genome browser [17,19,20]. The phylogenetic tree underlying this alignment is shown in Figure 2. In this alignment, 75% of the mouse genome was aligned with at least one other species. Using a patched version of the script rnazWindosws.pl, which comes with RNAz [21], we sliced each alignment block into overlapping windows (120 alignment-columns long, overlapping by 100 columns) and prefiltered the blocks to remove sequences with too many gaps using the *–max-gap=0.7* option. Since RNAz does not work well with alignments containing more than 10 species, we then generated up to five different samples of 10 species using the *–num-samples=5* option of rnazWindosws.pl whenever too many species were present in a window. Finally, windows or samples were filtered to remove those with less than a 50-column length or a very atypical mean pairwise identity (MPI) (below 40% or exactly 100%). We did not filter soft-masked genomic regions. These filtering criteria are less strict than the default parameters, since further steps of our pipeline would work as additional filtering steps. Blocks with a length below 50 nucleotides made up around 25% of the aligned region, while the other filters rarely kicked in.

These windows were scored with RNAz on both strands using a dinucleotide shuffled background model and a cutoff for RNA class probability of 0.5. Overlapping or “book-ended” RNAz hits were grouped into loci, irrespective of the strand. Whenever loci extended over multiple alignment blocks, we tried to merge these alignment blocks by either fetching up to 20 missing nts directly from the genome or by removing some species from the alignment. We merged when: at least two species would remain in the alignment, at most five species were in the initial blocks, and the resulting merged block would be below 400 alignment columns. If merging was not possible, the locus was split into two or more loci at the boundaries of the alignment blocks.

Next, we performed structural realignments and boundary prediction using LocARNA-P. Since the boundary prediction will always shrink the alignment, we added 20 nts of flanking genomic regions at both sides of the alignment for each species. Structural realignment was performed with LocARNA-P using the suggested parameters from the manual [37]. Each locus was realigned separately on both strands. Less than 100 realignment jobs did not finish after 2 weeks and were killed. LocARNA-P’s boundary prediction was performed, allowing for multiple reliably aligned regions per locus. All such regions which at least partly overlapped the original locus without flanking regions were merged by including the nucleotides in between. These regions of improved boundaries were again passed through rnazWindows.pl for sampling in cases where there were more than 10 species per alignment, and for removing loci with fewer than 50 alignment columns (but without cutting the region into sliding windows), and reclassified on their respective strands using RNAz (with the decision model for structural alignments.)

The set of raw loci contains all regions with at least one sample (if sampling of species was performed) having a RNAz score above 0.5 in the second classification step.

### 4.2. Estimation of False Discovery Rate

We split the original input alignment into chunks of 100 alignment blocks and shuffled every fifth chunk using multiperm [38]. This way, we ensured that our random reference was equally spread through the genome while keeping the majority of alignment blocks next to each other, retaining the possibility of merging alignment blocks as in the original pipeline. After the initial shuffling, the entire pipeline was repeated for the plus strand without modification, which included fetching unshuffled flanking regions.

The final loci in the shuffled alignment have virtually no overlap with the loci in the unshuffled alignment, which shows that shuffling efficiently destroys the original structure

### 4.3. Creation of a High-Confidence Set

Since a lot of data are available for genome-wide screens, we can estimate the false discovery rate for subsets of the alignment while still retaining enough data points to avoid overfitting. Unsurprisingly, the FDR of the raw loci was strongly dependent on the number of species in the input alignment (see Supplementary Figures S2 and S3), which is why we created a set of high-confidence loci with the following cutoffs:

- Loci with only two species were only included if the RNAz class probability is above 0.99.
- For loci with 3–10 species, we used a score cutoff of 0.9.
- For loci with more species, where samples were drawn, we required that at most one sample has a score below 0.5, and at least one sample has a score above 0.90.

### 4.4. Testing the Sensitivity Using Small Noncoding RNAs

To test the sensitivity of our screen, small ncRNA types ‘snoRNA’, ‘scaRNA’, ‘snRNA’, and ‘miRNA’ were extracted from the GENCODE mouse M17 annotation (assembly GRCm38) and intersected with RNAz loci using bedtools. miRNAs were restricted to loci listed in miRBase [39,40], again by intersecting M17 and miRBase miRNA annotations, resulting in 1185 out of 2265 genes. To assign GENCODE M17 snoRNAs and scaRNAs, database files from RNAcentral v9 [41] and Rfam 13.0 [42] were intersected with GENCODE transcript annotation for gene types snoRNA and scaRNA. For 1535 out of 1545 snoRNAs listed in GENCODE M17, we found an RNAcentral entry; for 1327, there was an Rfam family assigned. In total, the set contains 420 CD-box and 907 HACA-box snoRNAs. We processed the GENCODE genes of type ‘scaRNA’ the same way to ensure data quality was equal to CD-box and HACA snoRNA. All 51 scaRNAs in M17 annotations are listed in RNAcentral, and 40 were linked to an Rfam family. snRNAs were further subdivided into families, resulting in 965 genes. In total, our high-quality set of small RNAs of the classes miRNA, snRNA, scaRNA, and snoRNA consists of 3517 genes. The tRNA loci annotations were created using tRNA-Scan-SE [30]. Small RNA annotations and RNAz loci were intersected using bedtools.

### 4.5. Data Analysis

Data analysis was performed using bedtools [43], and custom Perl, python, shell, and R scripts. Results were plotted using matplotlib [44] and ggplot [45]. Information about repeat names used in RepeatMasker was obtained from RepBase [46,47]. The enrichment of repeat regions was calculated as the fraction of nucleotides of the high-confidence hits which overlap the repeat annotation, divided by the fraction of nucleotides in the input windows which overlap the annotation. Here, overlaps were calculated irrespective of the strand. Only repeat classes with more than 1000 annotated loci were used. Similarly, the repeat coverage enrichment for the high-confidence hits with respect to input alignments was calculated as the fraction of nucleotides of the high-confidence hits covered by a specific repeat class, divided by the the fraction of nucleotides in the input windows for that repeat class.

The false discovery rate (FDR) was calculated as the fraction of nucleotides predicted in the random control multiplied by 5 (because only 20% of the genome was shuffled), divided by the number of nucleotides in the true predicted loci. This estimation was only performed for the plus strand and assumed to be the same on the minus strand. For the subgroups of the genomic alignment based on MPI or the number of species, a factor of 5 was confirmed to be almost exactly the fraction of nucleotides in the initial windows of the unshuffled genome over the number of nucleotides in the initial windows of the random control.

### 4.6. Genomic Coverage of RNAz Hits

To perform the genomic partitions, we used the transcript biotype annotations available from the GENCODE release M17 [48] and described there [49]. We removed multiple identities of the genomic intervals in overlapping genes and transcript isoforms by projecting the annotated features of the gene onto the genome in a hierarchical fashion on five different levels: genomic (genic, intergenic), genic (exonic, intronic), exonic (noncoding, mRNA, other), mRNA (5’-UTR, 3’-UTR, CDS) and noncoding (lncRNA, sncRNA).

The annotated transcript biotypes considered to represent the superclass of mRNA, lncRNA, sncRNA (short non-coding RNA), and other can be found in the Supplementary Table S3.

In order to see if the coverage of RNAz hits is enriched in a specific genomic part, we intersected all the raw RNAz hits (with *p* > 0.5) against the respective genomic parts (minimum of 1 nucleotide overlap), and compared the fraction of total nucleotides of RNAz hits in a given part against the fraction of nucleotides of the genome in this part that are in alignments and used as input to RNAz. If the RNAz hits were evenly distributed in all genomic partitions, then the fraction of total nucleotides of RNAz hits in a given part should be the same as the fraction of nucleotides of the aligned genome (used as input to RNAz) in this part.

The genomic partitions and RNAz hit coverage enrichment analysis were performed using the bedops [50] program package v2.4.26 and custom R scripts, respectively.

### 4.7. Gene Ontology Enrichment Analysis

In order to identify if the RNAz hits were particularly enriched for the genes annotated as a specific ontology term, we downloaded the GO annotations from the Ensembl database [51] using biomaRt [52] services in R. For each gene with a particular ontology term, we then selected all the protein-coding transcript isoforms and collapsed their genomic coordinates considering the annotations for the 3’-UTR, 5’-UTR, and CDS. Now that we had the gene-specific unique genomic coordinates for UTRs and CDS, we intersected them against the RNAz hits.

Next, we computed the GO-specific enrichment of RNAz hits (mapped to 3’-UTR, 5’-UTR, CDS) by comparing: (1) the proportions of coverage of RNAz hits (minimum of 1 nucleotide overlap) in genes annotated as a given GO term divided by the total nucleotides covered by RNAz hits in all GO terms, against the total length of genes in this GO term divided by the total length of genes in all GO terms; (2) the proportions of the total number of genes with at least one RNAz hit (minimum overlap of 50% nucleotides of RNAz hit) for a given GO term divided by the total No. of genes with at least one RNAz hits in all GO terms, against the total number of genes in this ontology term divided by the total number of genes in all ontology terms.

The enrichment analysis was performed separately for the three extensive ontologies—molecular function, biological process, and cellular component. *p*-values were computed using Fisher’s exact test and were adjusted for multiple testing using the Benjamini–Hochberg procedure, as implemented in the *p.adjust* function in R, with a false discovery rate of 5%.

### 4.8. Covariance Models

In order to find genomic loci that show secondary structures strongly similar to our RNAz hits, covariance models (CMs) were built, using the cmbuild and cmcalibrate programs from the INFERNAL toolsuite [11]. For all loci, corresponding CMs were built using their multiple sequence alignment with LocARNA-P predicted boundaries, alongside its consensus RNA secondary structures as calculated by RNAalifold-r from the Vienna RNA package [53].

Since construction and subsequent calibration of CMs from a multiple sequence alignment and its consensus secondary structure is a computationally highly resource-consuming task, we preselected a subset of loci derived from the total set of high-confidence loci for CM building and subsequent screening against the full mouse genome. In order to be considered for a CM, a high-confidence locus was required to have (1) at least three sequences, (2) a total number of columns between 50 and 200. Enforcing constraint 1 ensured that all multiple sequence alignments showed at least a minimum amount of sequence variation and excluded non-informative alignments with only two species. Imposing restrictions on alignment length was necessary, since very small loci usually carry structural features that can be found multiple times throughout the genome and therefore render the CM unspecific, whereas the building of CMs from long loci takes a disproportionate amount of computational time and was therefore omitted.

Subsequent to successful calibration, using INFERNAL’s cmpress and cmscan utilities, the CMs were used to screen both strands of the entire mouse genome (mm10) for structurally similar loci using default parameters. For further downstream analysis, only hits below an *E*-value significance cutoff of $10^{-5}$ were considered. Merged RepeatMasker repeat annotations were intersected with the source loci of covariance models to generate a set of at least 60% repeat-free CMs. The distribution of these CMs across different genome partitions is shown as Venn diagram plotted using the Venn library in R [54].

### 4.9. Building a UCSC Genome Browser Trackhub

We built a UCSC genome browser track hub [55] with three tracks (RNAzGenome, RNAzRepeats, CMstat), further divided into subtracks (total 129). The high-confidence RNAz hits and cmsearch hits with *E*-value $\leq 10^{-10}$ were selected. For the track ‘RNAzGenome’, we projected gene elements onto the genome in a hierarchical fashion (genic, intergenic; exonic, intronic; CDS, 5’-UTR, 3’-UTR) using custom Perl scripts and bedtools. RNAz hits were intersected with genome partitions (*bedtools intersect-f 0.6*). For the track ‘RNAzRepeats’, repeat classes [27] were merged and intersected with RNAz hits (*bedtools intersect -f 0.6*). The cmsearch hits (‘CMstat’ track) were subdivided based on cmsearch *E*-values and the number of hits within each of these sets; bedplus files with RNAz hits were converted to bigBed format [56]. The trackhub file is freely available for noncommercial use at http://rna.tbi.univie.ac.at/trackhubs/#RNAz.

## Figures and Tables

**Table 1:** Enrichment/ depletion of high-confidence RNAz loci in annotated repeats for the repeat classes defined by RepeatMasker/ RepBase. Enrichments are measured in nucleotides covered by RNAz loci in the repeat class, relative to the total length of the repeat class covered by input alignments.

Repeat Class	Enrichment	# Hits Overlapping This Repeat	Coverage
LINE	0.91	16,736	0.0093
Low	1.07	3031	0.0110
Other	1.21	648	0.0124
DNA	1.36	3275	0.0139
rRNA	1.42	18	0.0145
scRNA	1.52	97	0.0156
LTR	1.78	26,720	0.0183
Unknown	1.85	256	0.0190
SINE	1.93	31,777	0.0197
tRNA	3.12	99	0.0320
snRNA	3.64	55	0.0373
Simple	5.49	24,228	0.0563
Satellite	6.53	2264	0.0670

**Table 2:** Enrichment of high-confidence RNAz hits in the 3’-UTR for Gene Ontology (GO) terms describing Molecular Function. **COV_E**: Enrichment in terms of nucleotides coverage. **CNT_E**: Enrichment in terms of counts. ***p*****-value** is calculated for the enrichment in counts. All GO terms with *p*-value < 0.05 are listed.

GO Terms	COV_E	CNT_E	*p*-Value
**binding**
protein binding	1.12713	1.31491	$8.8601\times 10^{-11}$
DNA binding	1.32868	1.55267	$9.3170\times 10^{-6}$
nucleic acid binding	1.54472	1.72894	$7.7324\times 10^{-7}$
RNA polymerase II proximal promoter sequence-specific DNA binding	1.44296	1.97414	$1.2507\times 10^{-3}$
mRNA 3’-UTR binding	1.68589	3.38916	$4.1919\times 10^{-2}$
metal ion binding	1.10881	1.25211	$1.1106\times 10^{-2}$
**transcription regulator activity**
transcription regulatory region DNA binding	1.60143	2.18793	$4.9220\times 10^{-3}$
transcriptional activator activity, RNA polymerase II proximal promoter sequence-specific DNA binding	1.38554	1.93798	$2.9325\times 10^{-2}$
transcription factor binding	1.54446	1.81153	$4.6530\times 10^{-2}$
DNA binding transcription factor activity	1.22179	1.58048	$5.7769\times 10^{-3}$
phosphoprotein phosphatase activity	1.34633	2.34482	$4.5489\times 10^{-2}$
**cellular response to stimulus**
G-protein coupled receptor activity	0.61850	0.57150	$1.2113\times 10^{-3}$
olfactory receptor activity	0.56060	0.48937	$1.9559\times 10^{-3}$
signal transducer activity	0.63878	0.60847	$2.6432\times 10^{-3}$

**Table 3:** Sensitivity analysis. The columns **annotated** contain the absolute number of annotated transcripts belonging to this class. The column **filtered** gives the number of transcripts from this group that are at least 80% within a continuous region of the filtered alignment. The column **found** gives the number of RNAs from the last column that are at least 60% overlapped by a single locus on the strand at which they are annotated. (In addition to the reported numbers, there are a few cases were a locus is only found on the reverse complement of an annotated RNA, or an RNA not 80% inside the filtered alignment is found by our screen). The **percent** value is calculated based on the columns “found” and “filtered”. miRNA: micro RNA; snRNA: small nuclear RNA, snoRNA: small nucleolar RNA, scaRNA: small Cajal body-specific RNA; tRNA: transfer RNA

RNA Class	Annotated	Filtered	Found	Percent
**miRNA**	1183	450	201	45%
**snRNA**	965	298	19	6%
… U1	134	64	4	6%
… U11	4	2	1	50%
… U4/U4atac	20	4	2	50%
… U5	11	7	3	43%
… U6/U6atac	708	175	0	0%
… U7	51	34	8	24%
**snoRNA**	1327	687	89	13%
… CD-box	420	191	12	6%
… HACA-box	907	496	77	16%
**scaRNA**	40	12	3	25%
… HACA-box	30	10	3	30%
**tRNA**	4488	1079	39	4%

## Supplementary Materials

The following are available online at http://www.mdpi.com/2073-4425/9/8/392/s1. Reference [57] is cited in the Supplementary Materials. Figure S1: Input alignment characteristics, Figure S2: False discovery rate as a function of the number of species, Figure S3: False discovery rate as a function of sequence sampling protokol, Figure S4: False discovery rate for alignments with 2 species, Figure S5: False discovery rate as a function of the mean pairwise identity, Figure S6: Example of a locus in a repeat region, Figure S7: Example for an enriched 3’-UTR element, Figure S8: Distribution of repeats over biotypes, Table S1: Enrichment for Gene Ontology terms describing Biological Process, Table S2: Enrichment for Gene Ontology terms describing Cellular Component, Table S3: Classification of biotypes into sncRNA, lncRNA and other, Table S4: Example for an enriched 3’-UTR Element.
