# Supplementary material for: RNA Structure Elements Conserved between Mouse and 59 Other Vertebrates
Source: Genes (Basel). 2018 Aug 1;9(8):392. doi: 10.3390/genes9080392 (PMC6116022; doi:10.3390/genes9080392)
Supplement: Supplementary file 1 [file genes-09-00392-s001.pdf]

Supplementary Materials: Conserved RNA structures in the mouse genome

Bernhard C. Thiel, Roman Ochsenreiter, Veerendra P. Gadekar, Andrea Tanzer, Ivo L. Hofacker

1. The input alignment

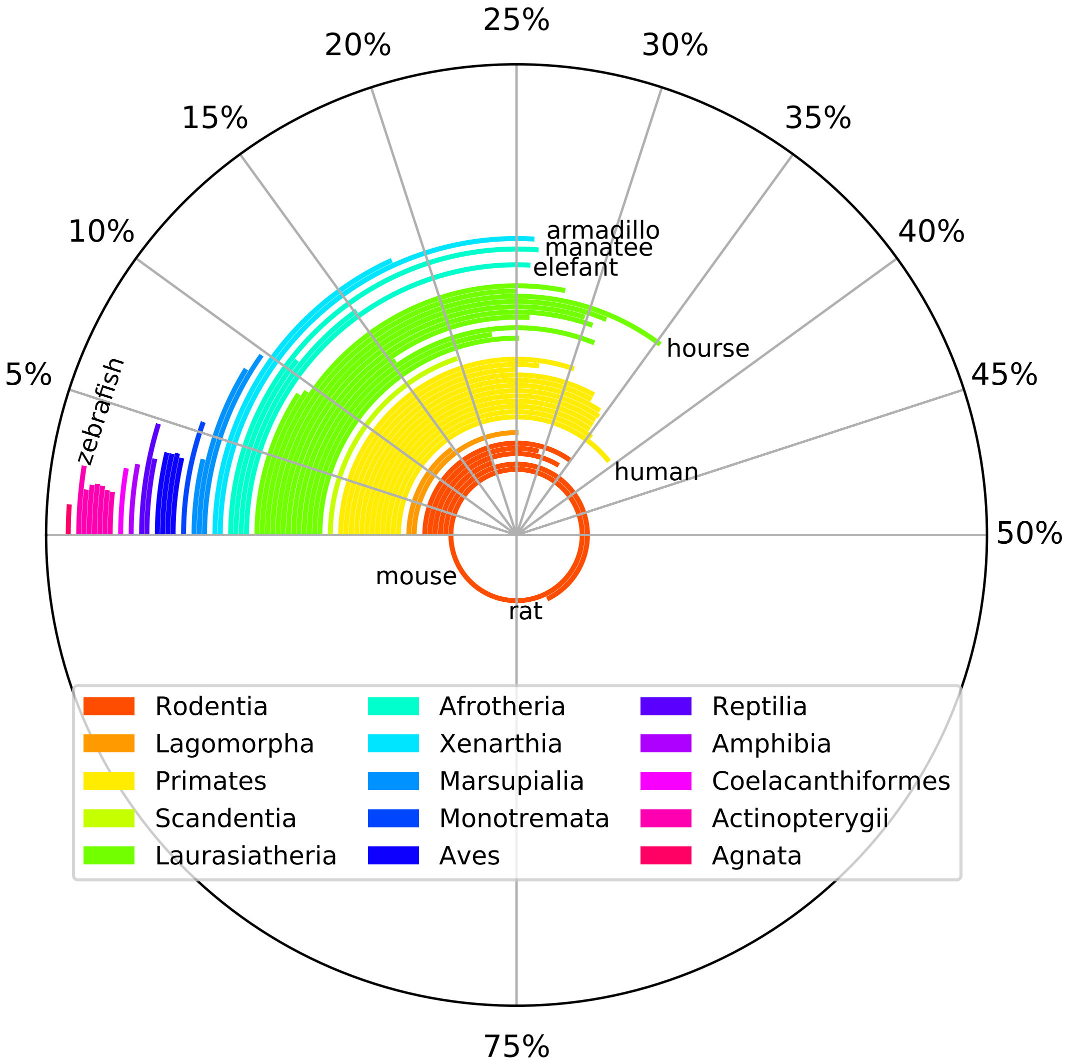

Figure S1. Input Alignment Characteristics. For each species, the number of nucleotides from mouse that align with this species is plotted as a fraction of the mouse genome length.

As shown in Figure S1, well studies species with high quality genome assembly are apparently better aligned with mouse in the 60 way multiz input alignment than species with lower genome quality. In particular, we notice that a larger fraction of the mouse genome was aligned with human than with rodents (except for rat). Furthermore there are even alignment blocks which only contain mouse and zebrafish.

This additionally impairs interpretation of our results in terms of conservational deepness.

2. The False Discovery Rate (FDR) as a function of the input alignment

Although the RNAz class probability was calibrated to work as consistently as possible for different input alignments, our complex pipeline featuring a realignment step caused the final FDR to be highly dependent on different features of the input alignment. In a recent comparable screen by Seemann et al.[1] the calibration of the cutoff of their CMfinder score was done in a GC-dependent manner based on the FDR estimation. Since the RNAz class probability is reasonable well calibrated for GC content and overfitting has to be avoided, we choose the RNA class probability cutoff only based on the number of species in the alignment.

For the raw RNAz loci, the dependency of the FDR on the number of species (Figure S2) is very strong. The lowest FDR is observed for alignments with 3 to 10 species, where the FDR lies between 20% and 30%. It is known that RNAz due to its dependency on RNAalifold has lower specificity if only 2 species are part of the input alignment, an effect which could not be compensated during the SVM calibration. For more than 10 species, we start to sample subsets of 10 species which will be classified by RNAz. Since a raw locus requires only one of 6 samples to be classified as RNA, we get a higher FDR if we sample. Furthermore, as the number of species in the input alignment increases, we can create more diverse samples, thus further increasing the chance to pick up random noise as signal.

We then looked at the FDR for alignments with only two species as a function of RNA class probability cutoff calculated by RNAz (Figure S4). To achieve a FDR compareable to that of alignments with 3 to 10 species and a score cutoff of 0.5, we have to use a score cutoff of 0.99 for alignments with exactly two species.

For more than 10 species in the alignment, we observe that the number of samples which have to be classified as RNA by RNAz has an effect on the FDR (see Figure S3) that outweighs the effect of the score cutoff. If we count everything as hit where at least one or two samples are classified as RNA, we have a high FDR. By requiring at least 5 samples to be RNAz positive, we achieve a better FDR while retaining enough hits.

This leads to the creation of a set of high confidence loci used in the main text.

Due to the realignment steps, the FDR is lowest for loci with low mean pairwise identity (MPI), as shown in Figure S5).

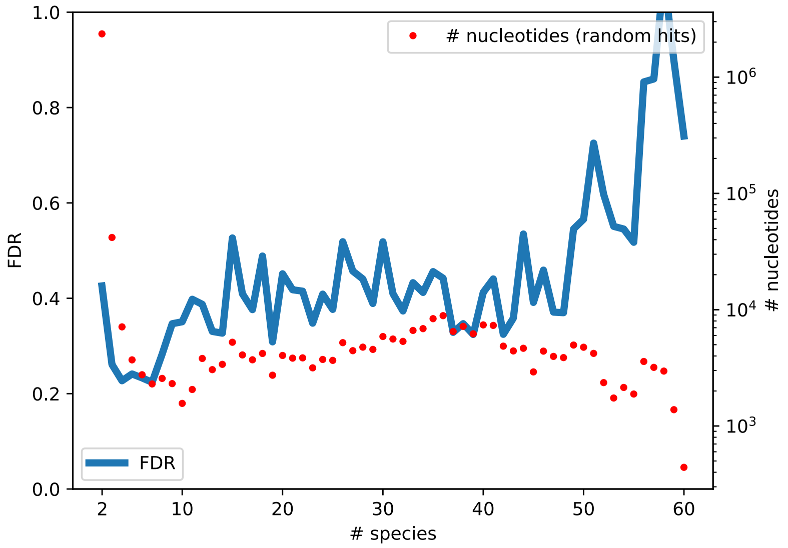

Figure S2. The false discovery rate (FDR) of the raw loci as a function of the number of species in the input alignment. The number of RNAz positive nucleotides in our random control for each class is plotted on the secondary axis to give an intuition of the statistical robustness of these results (note that a locus has around 200 nucleotides on average)

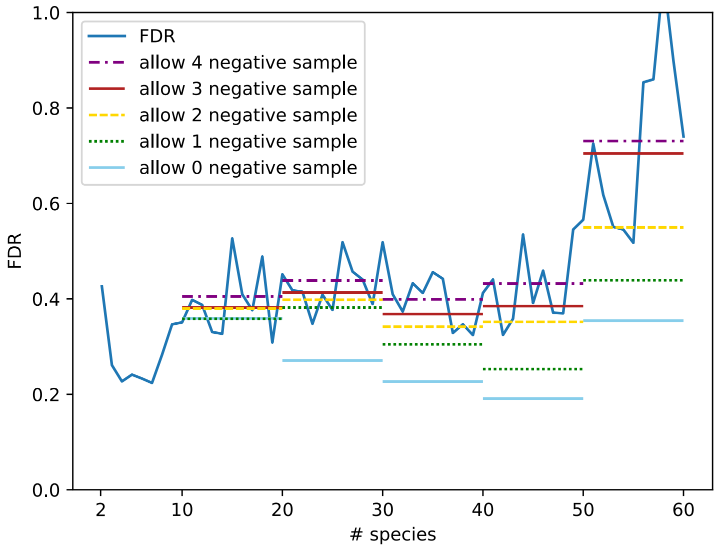

Figure S3. The false discovery rate (FDR) as a function of the number of species with different treatment of loci where sequences were sampled. Due to the limited number of data, we bin the data.

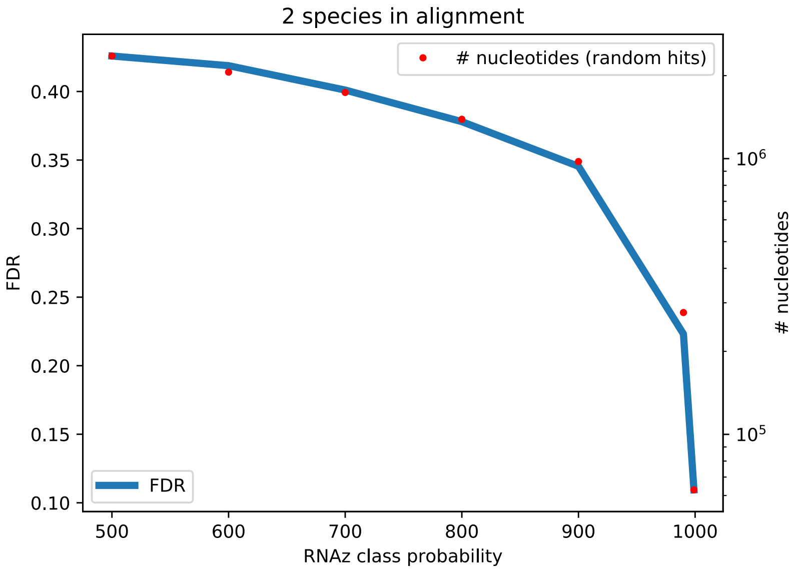

Figure S4. The false discovery rate (FDR) for alignments with 2 species as a function of the RNA class probability cutoff (as promille).

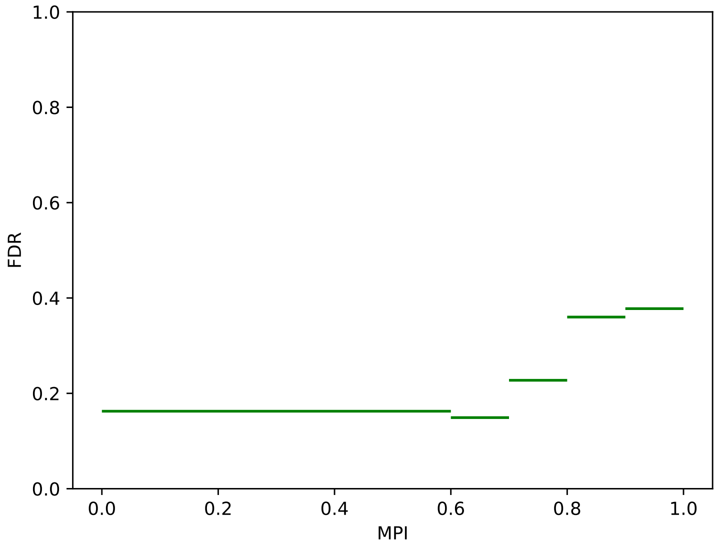

Figure S5. The false discovery rate (FDR) of the high confidence loci as a function of the mean pairwise identity (MPI), created using binned data.

3. Enrichment of high confidence RNAz hits in the 3′-untranslated region (3′-UTR) for Gene Ontology (GO) terms describing biological process and cellular component

Table S1. Enrichment of high confidence RNAz hits in the 3′-untranslated region (3′-UTR) for Gene Ontology (GO) terms describing biological process. **COV_E**: Enrichment in terms of nucleotides coverage. **CNT_E**: Enrichment in terms of counts. ***p*-value** is calculated for the enrichment in counts.

GO terms	COV_E	CNT_E	p-value
**gene expression**			
transcription, DNA-templated	1.38888	1.74526	1.9229×10^{-10}
regulation of transcription, DNA-templated	1.39315	1.64643	1.9229×10^{-10}
positive regulation of transcription from RNA polymerase II promoter	1.43190	1.85313	1.5975×10^{-7}
negative regulation of transcription from RNA polymerase II promoter	1.41704	1.87274	2.8419×10^{-5}
positive regulation of transcription, DNA-templated	1.40613	1.93409	2.8026×10^{-4}
transcription from RNA polymerase II promoter	1.47576	2.02047	1.9163×10^{-4}
positive regulation of gene expression	1.37205	1.87336	3.2757×10^{-2}
**nervous system process**			
nervous system development	1.10095	2.01435	4.0771×10^{-3}
axon guidance	1.39357	2.56867	4.3024×10^{-2}
**response to stimulus**			
response to stimulus	0.59064	0.52603	1.3964×10^{-2}
detection of chemical stimulus involved in sensory perception of smell	0.56807	0.50006	1.3964×10^{-2}
sensory perception of smell	0.55678	0.50119	1.1933×10^{-2}
G-protein coupled receptor signaling pathway	0.63762	0.60856	1.0636×10^{-2}
**metabolic process**			
oxidation-reduction process	0.65029	0.46971	3.2757×10^{-2}

Table S2. Enrichment of high confidence RNAz hits in the 3′-UTR for GO-terms describing cellular component. **COV_E**: Enrichment in terms of nucleotides coverage. **CNT_E**: Enrichment in terms of counts. ***p*-value** is calculated for the enrichment in counts.

GO terms	COV_E	CNT_E	p-value
**intracellular part**			
cytoplasm	1.11736	1.20829	4.9443×10^{-4}
cytosol	1.16312	1.31489	4.9443×10^{-4}
cytoplasmic stress granule	3.01697	4.24796	5.4124×10^{-3}
**nuclear part**			
nucleus	1.24812	1.37075	4.1530×10^{-12}
nucleoplasm	1.34421	1.58915	3.6208×10^{-9}
**synapse**			
postsynaptic density	1.20675	2.14592	3.0239×10^{-2}
**extracellular**			
extracellular region	1.01198	0.60685	1.5071×10^{-3}

4. Example of a locus in a repeat region

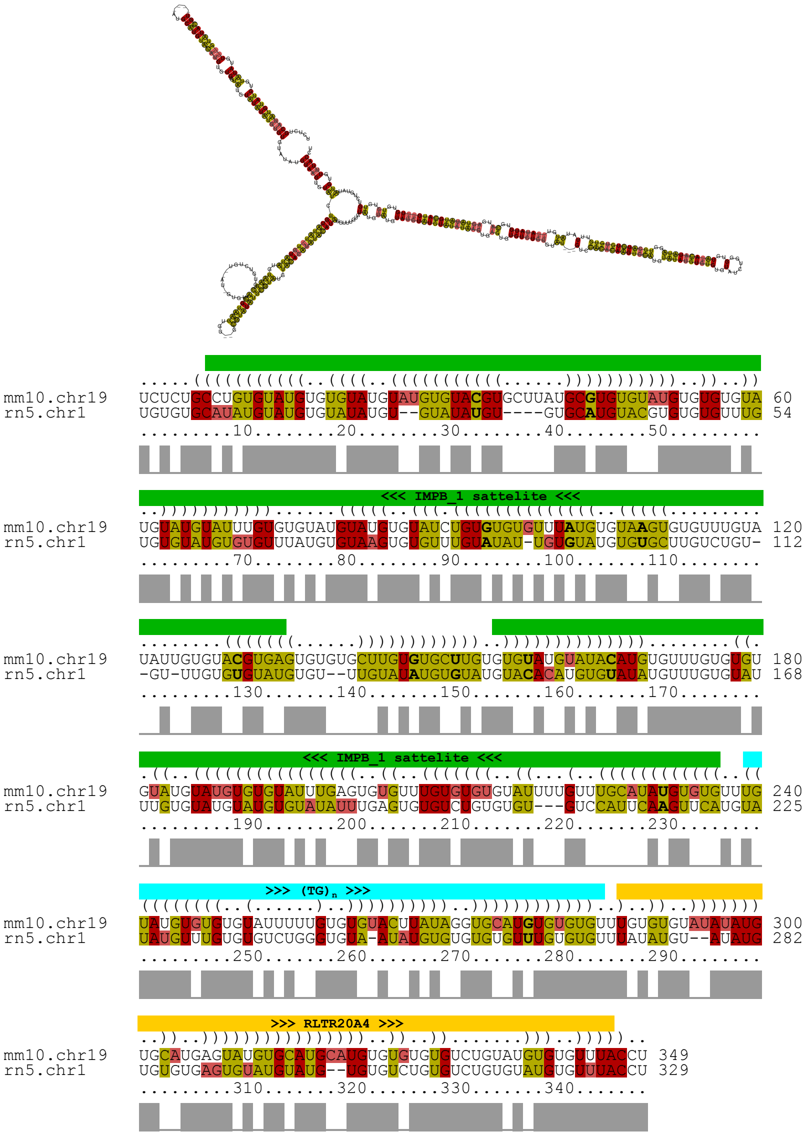

Figure S6. Example of a structure with support from covarying basepairs (bold letters in the alignment) that overlaps regions masked as IMPB_1 satellite repeat[2] and RLTR20A4[3] (Long terminal repeat of retrovirus-like element) located at chromosome 19, nucleotide 31846601 to 31846950 on the forward strand, locus1761533.. The corresponding rat sequence is annotated as two stretches of RLTR20A4 seperated by a simple TG repeat.

5. Classification of biotypes into sncRNA, lncRNA and other

Table S3. Transcript biotype annotations as per Ensembl Release 92 (April 2018). See [4] for the definition of the used biotypes. We classify noncoding biotypes into 3 categories: long noncoding RNA (lncRNA), short noncoding RNA (sncRNA) and other. This classification was used for Figures 3 and 5 in the main article.

Class	Biotypes	Number	Total
mRNA	protein_coding	57047	57047
lncRNA	processed_transcript	15315	27964
lncRNA	lincRNA	8224	27964
lncRNA	antisense	4164	27964
lncRNA	bidirectional_promoter_lncRNA	256	27964
lncRNA	3prime_overlapping_ncRNA	3	27964
lncRNA	macro_lncRNA	2	27964
sncRNA	miRNA	2202	5500
sncRNA	snoRNA	1507	5500
sncRNA	snRNA	1383	5500
sncRNA	rRNA	354	5500
sncRNA	scaRNA	51	5500
sncRNA	sRNA	2	5500
sncRNA	scRNA	1	5500
other	retained_intron	20517	44633
other	processed_pseudogene	9125	44633
other	nonsense_mediated_decay	6593	44633
other	TEC	3189	44633
other	unprocessed_pseudogene	2599	44633
other	misc_RNA	566	44633
other	sense_intronic	347	44633
other	IG_V_gene	301	44633
other	transcribed_processed_pseudogene	273	44633
other	transcribed_unprocessed_pseudogene	247	44633
other	TR_V_gene	194	44633
other	IG_V_pseudogene	155	44633
other	pseudogene	95	44633
other	polymorphic_pseudogene	93	44633
other	TR_J_gene	70	44633
other	sense_overlapping	53	44633
other	TR_V_pseudogene	34	44633
other	non_stop_decay	25	44633
other	ribozyme	22	44633
other	unitary_pseudogene	21	44633
other	IG_C_gene	21	44633
other	IG_D_gene	19	44633
other	transcribed_unitary_pseudogene	14	44633
other	IG_J_gene	14	44633
other	translated_processed_pseudogene	12	44633
other	TR_C_gene	10	44633
other	TR_J_pseudogene	10	44633
other	IG_LV_gene	4	44633
other	TR_D_gene	4	44633
other	IG_D_pseudogene	3	44633
other	IG_pseudogene	2	44633
other	IG_C_pseudogene	1	44633

6. Example for an Enriched 3′-UTR Element

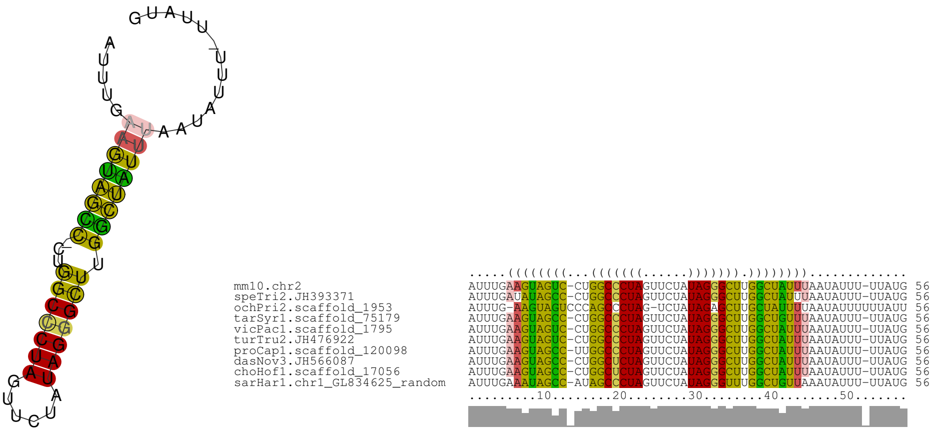

Figure S7. Consensus structure and alignment of a locus from chromosome 2. Structurally similar hits found by a Cpvariance Model (CM) made from this locus are enriched in 3′-UTRs.

Table S4. Genes with hits derived from the locus shown in Figure S6. T The two most significant hits map to 3′UTR's of genes.

Gene ID	Chr.	Coordinates	Gene Description	E-value
ENSMUSG00000027598	2	155226397-155226452	**3′UTR, Source of CM.** E3 ubiquitin-protein ligase Itchy	10^{-10}
ENSMUSG00000067285	5	42216341-42216396	**3′UTR**. predicted gene 16223	10^{-7}
ENSMUSG00000004530	5	113900052-113900107	**Intron.** Coronin-1C	6.3×10^{-3}

7. Distribution of repeats over biotypes

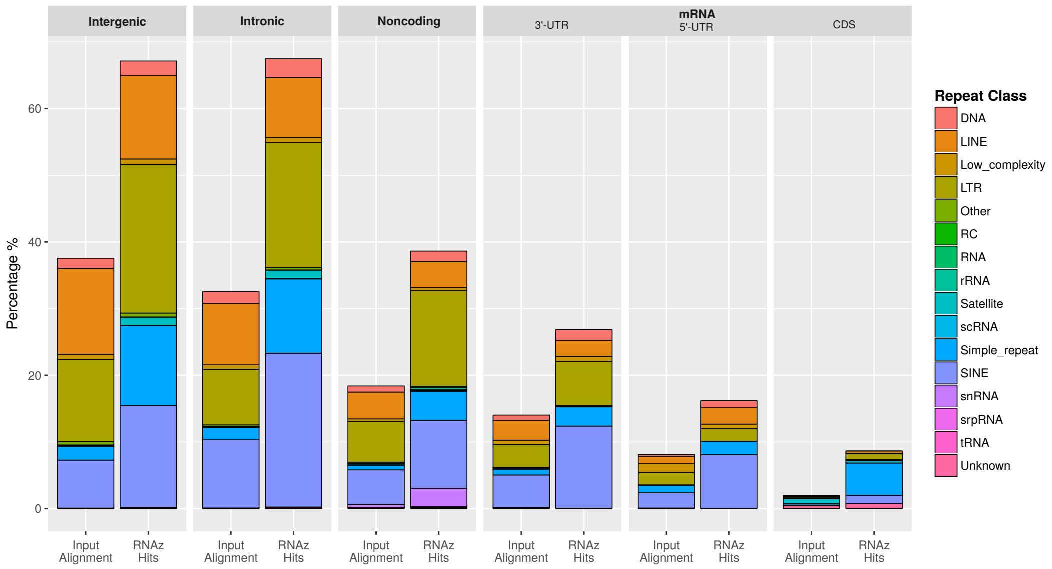

Figure S8. Distribution of repeats over the genome and RNAz high confidence hits.

8. References

1. Seemann, S.E.; Mirza, A.H.; Hansen, C.; Bang-Berthelsen, C.H.; Garde, C.; Christensen-Dalsgaard, M.; Torarinsson, E.; Yao, Z.; Workman, C.T.; Pociot, F.; et al. The identification and functional annotation of RNA structures conserved in vertebrates. *Genome Res.* **2017**, *27*, 1371–1383.
2. Tetuev, R.; Nazipova, N.N. Consensus of repeated region of mouse chromosome 6 containing 60 tandem copies of a complex pattern. *Repbase Rep.* **2010**, *10*, 776.
3. Jurka,J. Long terminal repeats from Murinae. *Repbase Rep.* **2009**, *9*, 1462.
4. Gene/Transcript Biotypes in GENCODE & Ensembl. Available online: https://www.gencodegenes.org/gencode_biotypes.html (accessed on 30 July 2018).
